# Supplementary material for: Intergenic SNPs in Obstructive Sleep Apnea Syndrome: Revealing Metabolic, Oxidative Stress and Immune-Related Pathways
Source: Diagnostics (Basel). 2021 Sep 24;11(10):1753. doi: 10.3390/diagnostics11101753 (PMC8534397; doi:10.3390/diagnostics11101753)
Supplement: Supplementary file 1 [file diagnostics-11-01753-s001.zip › Supplementary File S2. SNPSnap Report.pdf]

SNPsnap

- [Home]
- [About]
- [Match SNPs]
- [Download]
- [FAQ]
- [Documentation]
- [Contact]
- [Feedback]

SNPsnap is now matching your SNPs

Please remember to cite SNPsnap

An email will be sent to **dantevavougios@hotmail.com** when the job is completed. You will be able to download the results as soon your job finish. You can download the results from the bottom of this page or via the below link.

Results URL: `https://www.broadinstitute.org/mpg/snpsnap/results/7de582dfc4bbfc77ef8dea443f383560/SNPsnap_test_run.zip`

If you browse back you will not be able to retrieve this site again. However, you will still receive an email notification about your job completion. ***Do not refresh this page, as it will duplicate your job****. That is, you will submit an additional identical job.*

You may experience that SNPsnap is 'hanging' in the initialization phase of a job. Please allow a few minutes for the job to get started before you submit a new one. Typical jobs finish within 10-60 minutes after which the download link becomes active and you can download the results.

Job Parameters

Job Status

Matching SNPs

complete

100%

Calculating match bias

complete

100%

Annotating Matched SNPs

complete

100%

Annotating Inputs SNPs

complete

100%

Clumping Input SNPs

complete

100%

SNPsnap Scores

SNPsnap score	Value	Rating
Insufficient-matches	50.00%	**Very Poor** Poor Ok Good Very Good
Match-size	16.99%	**Very Poor** Poor Ok Good Very Good

You may safely ignore the *Match-size* rating if the *Insufficient-matches* rating is better than Ok. See the documentation for more information.

Matching Bias

Genetic property	Mean input	Mean matched	Ratio (%)*
**Minor Allele Frequency**	7.50	7.44	100.75%
**Gene density**	4.50	3.94	114.12%
**Distance to nearest gene**	2842.00	2821.44	100.73%
**LD buddies**	14.50	13.51	107.34%

*Ratio defined as *Mean input/Mean matched*.
Requesting a large number of SNPs may lead to a systematic difference between genetic properties of input and matched SNPs indicated by a ratio deviating from 100%.
In order to obtain a ratio close to 100%, lessen the number of requested SNPs or tighten

the matching criteria.
See the documentation for more information.

Input Loci Independence

	Number of Loci
**Input Loci***	2
**Clumped Loci****	2

*Number of *valid* user input SNPs found in SNPsnap's SNP database.
**Number of loci after clumping based on user-specified parameters.

Your input SNPs are independent

See the file `input_snps_clumped.txt` for details on the clumped loci.
