# Supplementary material for: Intergenic SNPs in Obstructive Sleep Apnea Syndrome: Revealing Metabolic, Oxidative Stress and Immune-Related Pathways
Source: Diagnostics (Basel). 2021 Sep 24;11(10):1753. doi: 10.3390/diagnostics11101753 (PMC8534397; doi:10.3390/diagnostics11101753)
Supplement: Supplementary file 1 [file diagnostics-11-01753-s001.zip › Supplementary Materials S3. Gene Signature.pdf]

Supplementary Materials 3. Gene Signature (Genes and TFs)

1. VPS54
2. SERTAD2
3. RN7SL211P
4. LINC02576
5. SLC1A4
6. RAB1A
7. LINC01800
8. ACTR2
9. AFTPH
10. LOC100420338
11. SNRPE
12. LAX1
13. ZBED6
14. ZC3H11A
15. ARRB1
16. CEBPB
17. FOS
18. JUND
19. RAD21
20. HNRNPL
21. GATAD2A
22. CTCF
23. ZBTB33
24. PRDM10
25. ZNF629
26. TFE3
27. RFX1
28. LEF1
29. IKZF1
30. NFKBIZ
31. ZNF692
32. RCOR2
33. BACH1
34. JUND
35. LARP7
36. MTA2
37. ZNF143
38. RELA
39. ZIC2
40. PKNOX1
41. ZNF24
42. RXRB
43. MTA1

44. PATZ1
45. REST
46. NR2C1
47. RAD21
48. IRF2
49. IKZF2
50. ZNF740
51. BRD9
52. TRIM22
53. DPF2
54. HDAC1
55. GABPA
56. YY1
57. SOX6
58. CEBPA
59. EP300
60. BRCA1
61. KMT2B
62. MNT
63. EED
64. NRF1
65. SOX13
66. CEBPB
67. KLF9
68. MGA
69. ZBTB20
70. ETS1
71. GTF2E2
72. CBX3
73. SP1
74. UBTF
75. ZNF766
76. HMBOX1
77. RELB
78. SMARCC1
79. CHD2
80. POLR2A
81. MTA3
82. EGR1
83. CTBP1
84. TAF9B
85. GATAD2B
86. ZBTB2
87. NFE2
88. HES1

89. RNF2
90. HDGF
91. ZKSCAN8
92. ATF3
93. HDAC2
94. FOS
95. ZSCAN29
96. ZNF316
97. BCLAF1
98. NFRKB
99. CBFB
100. JUN
101. NR2C2
102. ZBTB11
103. MEF2D
104. CREB1
105. ZNF318
106. ZNF592
107. SMC3
108. E2F6
109. AFF1
110. ELK4
111. MBD1
112. MYC
113. ZKSCAN1
114. ZNF205
115. SMARCB1
116. TEAD1
117. ZNF423
118. EP400
119. MYRF
120. SIX5
121. TFDP1
122. KDM6A
123. NCOR1
124. FOSL1
125. MAFG
126. BCL11A
127. MAX
128. EHMT2
129. MAZ
130. CREM
131. MXI1
132. GTF2A2
133. MBD2

134. FOXP1
135. TBX3
136. NBN
137. ZSCAN9
138. CUX1
139. ZFHX2
140. FOSL2
141. ZNF792
142. SIN3A
143. MYNN
144. SAP130
145. TBP
146. ZNF644
147. ZNF189
148. NR2F1
149. JUNB
150. ZNF207
151. HDAC8
152. ZNF335
153. MIER3
154. BHLHE40
155. STAT1
156. GABPB1
157. ZNF395
158. SPI1
159. STAT3
160. RCOR1
161. ZNF511
162. ZGPAT
163. MXD4
164. SMARCA5
165. IRF4
166. ZFP69B
167. ELF1
168. ZNF584
169. DNMT3B
170. BATF
171. TCF7L2
172. GATAD1
173. SKIL
174. EBF1
175. KLF17
176. ZNF654
177. DRAP1
178. USF2

179. PHF20
180. HIC1
181. SMAD4
182. NR2F6
183. RB1
184. BCL6
185. NFE2L2
186. ADNP
187. ZNF589
188. HOMEZ
189. CHD4
190. KLF11
191. VEZF1
192. BRD4
193. TBL1XR1
194. FOXK2
195. ZFP64
196. NFIL3
197. IKZF5
198. ATF2
199. THAP11
200. ETV1
201. RARA
202. ID3
203. DMAP1
204. ZNF660
205. GLIS1
206. THRB
207. ARID4B
208. KDM1A
209. ETV4
210. KLF10
211. KLF16
212. ATF7
213. DIDO1
214. ZHX1
215. TEAD3
216. HMG20A
217. IRF9
218. SMARCA4
219. MITF
220. SP3
221. ZNF48
222. KLF1
223. GTF3C2

224. SMARCE1
225. MXD3
226. SOX5
227. TCF7
228. HSF1
229. ZHX2
230. ZNF580
231. NFYC
232. HBP1
233. RLF
234. L3MBTL2
235. RBPJ
236. ZNF354B
237. NR4A1
238. ELF3
239. FOXA3
240. MLX
241. ZNF687
242. MIXL1
243. ZFX
244. ZNF148
245. TRIM24
246. CAVIN1
247. PPARG
248. CBFA2T3
249. ARID1B
250. ZBTB40
251. ZNF384
252. SP7
253. CC2D1A
254. BCL3
255. NR3C1
256. GLIS2
257. SP5
258. EMSY
259. ZNF639
260. RXRA
261. ZBTB8A
262. ETV6
263. XRCC5
264. PTTG1
265. ERF
266. GMEB1
267. RAD51
268. KAT8

269. ATF1
270. RERE
271. AHR
272. ZEB2
273. ERRA
274. ERRB
